# Deep learning-based estimation of axial length using macular optical coherence tomography images

**DOI:** 10.3389/fmed.2023.1308923

**Published:** 2023-11-17

**Authors:** Jing Liu, Hui Li, You Zhou, Yue Zhang, Shuang Song, Xiaoya Gu, Jingjing Xu, Xiaobing Yu

**Affiliations:** ^1^Department of Ophthalmology, Beijing Hospital, National Center of Gerontology, Institute of Geriatric Medicine, Chinese Academy of Medical Sciences, Beijing, China; ^2^Graduate School of Peking Union Medical College, Beijing, China; ^3^Visionary Intelligence Ltd., Beijing, China

**Keywords:** optical coherence tomography, axial length, artificial intelligence, deep learning, Grad-CAM

## Abstract

**Background:**

This study aimed to develop deep learning models using macular optical coherence tomography (OCT) images to estimate axial lengths (ALs) in eyes without maculopathy.

**Methods:**

A total of 2,664 macular OCT images from 444 patients’ eyes without maculopathy, who visited Beijing Hospital between March 2019 and October 2021, were included. The dataset was divided into training, validation, and testing sets with a ratio of 6:2:2. Three pre-trained models (ResNet 18, ResNet 50, and ViT) were developed for binary classification (AL ≥ 26 mm) and regression task. Ten-fold cross-validation was performed, and Grad-CAM analysis was employed to visualize AL-related macular features. Additionally, retinal thickness measurements were used to predict AL by linear and logistic regression models.

**Results:**

ResNet 50 achieved an accuracy of 0.872 (95% Confidence Interval [CI], 0.840–0.899), with high sensitivity of 0.804 (95% CI, 0.728–0.867) and specificity of 0.895 (95% CI, 0.861–0.923). The mean absolute error for AL prediction was 0.83 mm (95% CI, 0.72–0.95 mm). The best AUC, and accuracy of AL estimation using macular OCT images (0.929, 87.2%) was superior to using retinal thickness measurements alone (0.747, 77.8%). AL-related macular features were on the fovea and adjacent regions.

**Conclusion:**

OCT images can be effectively utilized for estimating AL with good performance via deep learning. The AL-related macular features exhibit a localized pattern in the macula, rather than continuous alterations throughout the entire region. These findings can lay the foundation for future research in the pathogenesis of AL-related maculopathy.

## 1 Introduction

Axial length (AL) is a widely discussed parameter, significant not only for defining the eye’s refractive status but also due to its strong association with retinal and macular complications (1, 2). The excessive elongation of AL, often exceeding 26.0 mm, is the dominant cause of an increased risk of posterior segment complications, including vitreous liquefaction, choroidal atrophy, retinoschisis, macular hole, and macular choroidal neovascularization (3). These complications are vision-threatening and often result in irreversible and permanent vision damage if left untreated (4). In the past, it has not been clear whether there are pre-existing differences in macular structure among eyes with prolonged AL prior to the development of maculopathies, except a few studies have reported that AL was positively associated with central retinal thickness, but negatively associated with peripheral retinal thickness farther from the macula (5–8).

Artificial intelligence, specifically deep learning, has exhibited significant potential in medical imaging diagnosis and interpretation (9, 10). Deep learning allows systems to acquire predictive characteristics directly from an extensive collection of labeled images, eliminating the necessity for explicit rules or manually designed features (11). In recent research, deep learning models have been developed that demonstrate precise estimation of AL or refractive error using color fundus photographs (12–14). Additionally, Yoo et al. (15) have introduced a deep learning model that predicts uncorrected refractive error by utilizing posterior segment optical coherence tomography images, suggesting a potential association between AL and the sectional structure of the retina. Considering that a long AL is a significant risk factor for complications that can potentially impair vision, investigating the alterations in macular structure resulting from prolonged AL prior to the onset of maculopathies holds immense significance in guiding the clinical management and prognosis of patients with long AL eyes (4). However, the application of deep learning to estimate AL based on macular OCT images remains unexplored.

Gradient-weighted class activation mapping (Grad-CAM), a commonly employed approach for visualizing models, utilizes the gradient details that flows into the final convolutional layer of a convolutional neural network (CNN) to construct a heat map that unveils the pivotal regions that are most relevant for the decision-making process (16). This study aimed to assess the capability of macular OCT images to estimate ALs of eyes without maculopathy using deep learning algorithms and visualize the cross-sectional alterations in macular structure resulting from the prolonged AL using Grad-CAM.

## 2 Materials and methods

### 2.1 Study design and overview

The data of this study were retrospectively collected from patients who visited the Department of Ophthalmology at Beijing Hospital between January 2019 and October 2021 and were scheduled for cataract surgery. Patients included in the study were required to be aged 18 years or older and have undergone macular OCT examination and AL measurement. Eyes with evident macular abnormalities, such as macular edema, epiretinal membrane, macular hole, macular retinoschisis, and macular neovascularization, were excluded. Furthermore, images of poor quality were also excluded. The study followed the principles of the Declaration of Helsinki and received approval from the institutional review board at Beijing Hospital. Given the retrospective nature of the study, the requirement for written informed consent was waived.

In this study, OCT scans were acquired using the Spectralis OCT device (Heidelberg Engineering, Germany). Images scanned with a stellate scan model centered on the fovea were selected for model development. This scanning model comprises six scans that traverse the fovea, each spanning a length of 6 mm. Moreover, retinal thickness in various subfields was recorded using OCT. The macular region was divided into 9 subfields by employing three concentric circles centered on the fovea, with diameters of 1 mm, 3 mm, and 6 mm. The average thickness of the innermost ring defined the central retinal thickness (CRT). Furthermore, the inner (1–3 mm) and outer (3–6 mm) rings were subdivided into superior, nasal, inferior, and temporal subfields, designated as the parafovea and perifovea, respectively. AL measurements were obtained from the IOL Master 700 (Carl Zeiss, Germany).

### 2.2 Deep learning model and its training

**Figure 1:**
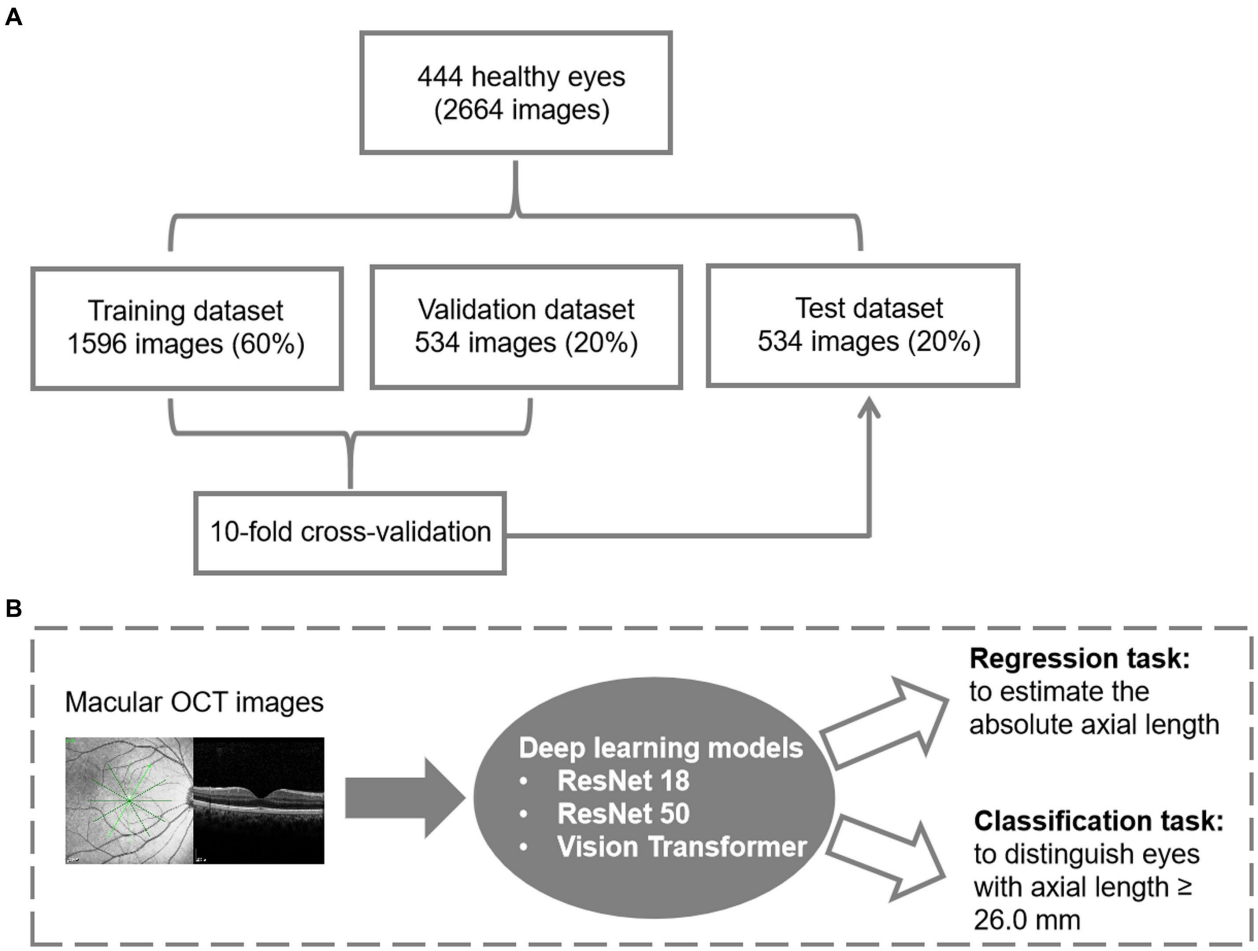
Datasets and the architecture of the deep learning model. **(A)** Data management for model development. **(B)** The flowchart for deep learning.

Figure 1 presents the data management and the flowchart for deep learning models in this study. Two classic CNN models, ResNet18 and ResNet50, along with a Transformer-based model called Vision Transformer (ViT), were introduced to establish the relationship. The detailed description of the models used in this study was presented in Supplementary material 1. In the ViT architecture, the number of encoder blocks was reduced to 6 to prevent overfitting. The input size for the vision transformer is fixed at 224 * 224 to ensure a fair comparison across all models. The SGD (Stochastic Gradient Descent) method serves as the optimizer for all three models. AL measurements obtained by IOL Master 700 (Carl Zeiss, Germany) served as the ground truth for AL prediction. The prediction task is divided into a regression task and a binary classification task by adjusting the dimension of the output result for comprehensive evaluation. To improve accuracy and efficiency, we implement a transfer learning strategy using models pretrained on ImageNet. The salient areas of the feature maps in the latter layers of these models are visualized using the Grad-CAM interpretability method, which illustrates the contribution of each pixel to the final decision.

During training, we employ multiple data augmentation methods to enhance the model’s generalization ability. The random resize crop strategy is used to capture different parts of the image with varying scales. Furthermore, horizontal flipping, color jittering, gamma transformation, and random Gaussian noise are applied to augment the training samples for OCT data. Eventually, we implement the normalization to scale the training and testing input from 0 to 1. To expand the dataset, each case is considered independent and equipped with 6 OCT B-scans. This allows us to formulate a dataset with 2,664 images. The data split ratio for training, validation, and testing was 6:2:2, and the split was randomized based on the AL. The training set and validation set were combined, and a 10-fold cross-validation was conducted to demonstrate the reliability of the methods. In the 10-fold cross-validation, the training instances are divided into 10 equally-sized partitions with similar class distributions. Subsequently, each partition is sequentially employed as the test dataset for the classifier generated using the remaining nine partitions.

### 2.3 Statistical analysis

The classification task utilized the cross-entropy loss function, and various metrics such as sensitivity, specificity, area under the receiver operating characteristic curve (AUC), and accuracy were calculated to evaluate performance. In the regression task, the MAELoss function was used as the loss function, and the mean absolute error (MAE) was used as the evaluation metric. The agreement between the actual and predicted AL was assessed using the Bland–Altman plot. The Y-axis represents the difference between the actual and predicted ALs, and the X-axis represents the average of the actual and predicted ALs. The mean difference (MD) and 95% limits of agreement (MD ± 1.96 standard deviations) were calculated to assess the agreement.

## 3 Results

### 3.1 AI models performance

**Figure 2:**
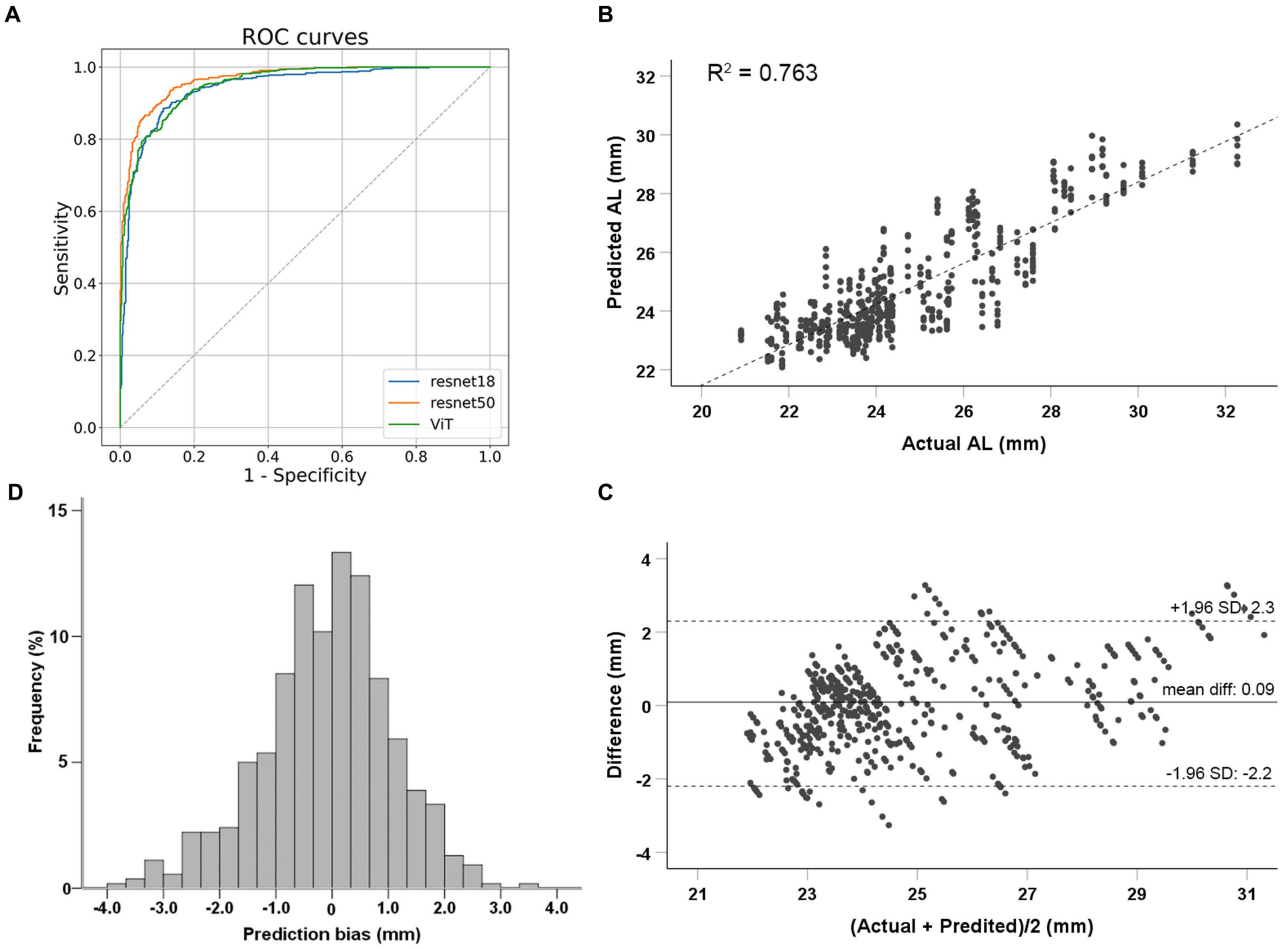
Performance evaluation of deep learning models. **(A)** Classification performance of deep learning models to identify eyes with axial lengths ≥26.0 mm in the test dataset. **(B)** Correlations between actual and predicted axial length using the ResNet 50 model. **(C)** Bland–Altman plots for the real and predicted axial length using ResNet 50 in test dataset. **(D)** Prediction bias frequency distribution.

Finally, a total of 2,664 images from 444 eyes (306 patients) were included in the model development. The mean age was 69.02 ± 10.37 years. Among 444 eyes, 113 eyes (25.5%) were high myopic without maculopathy (AL ≥ 26.0 mm). Finally, 266 eyes (1,596 images) were used for training (60%), 89 eyes (534 images) for validation (20%), and 89 eyes (534 images) for testing (20%). The mean age for the training, validation and testing set were 69.36 ± 10.52, 67.89 ± 10.65, and 69.21 ± 9.63 years, respectively. Demographic characteristics of each dataset are summarized in Table 1. Three models (ResNet 50, ResNet 18, and ViT) were developed for the binary classification task of distinguishing AL ≥ 26.0 mm from others. The 10-fold cross-validation results showed the robust performance and high discriminative power of all three models, as illustrated in Table 2. On the test dataset, ResNet 18, ResNet 50, and ViT achieved AUC (95% Confidence Interval [CI]) values of 0.918 (0.886–0.951), 0.929 (0.899–0.960), and 0.924 (0.892–0.955), respectively (as shown in Figure 2A). ResNet 50 and ResNet 18 had the same accuracy of 0.872 (95%CI, 0.840–0.899), which was the highest among the models. ResNet 50 also exhibited the highest performance, with a sensitivity of 0.804 (95%CI, 0.728–0.867) and specificity of 0.895 (95%CI, 0.861–0.923). Therefore, based on the classification results, particularly the AUC and accuracy, ResNet 50 was selected for further analyses.

**Table 1:** Summary of the demographical characteristics of training, validation, and test data sets.

	Training set	Validation set	Test set
No. of eyes	266	89	89
No. of images	1,596	534	534
Age, year	69.36 ± 10.52	67.89 ± 10.65	69.21 ± 9.63
Sex, male, *n* (%)	121 (45.5%)	43 (48.3%)	41 (46.1%)
AL, mm	24.67 ± 2.19	24.73 ± 2.19	24.73 ± 2.33
AL < 26 mm	199	66	66
AL ≥ 26 mm	67	23	23

AL, axial length.

**Table 2:** Performance of deep learning models for binary task (axial length ≥ 26.0 mm).

	Mean results of 10-fold cross validation				Test set (95% CI)			
	AUC	Accuracy	Sensitivity	Specificity	AUC	Accuracy	Sensitivity	Specificity
ResNet 18	0.908 ± 0.048	0.898 ± 0.042	0.807 ± 0.107	0.997 ± 0.008	0.918 (0.886, 0.951)	0.872 (0.840, 0.899)	0.783 (0.704, 0.848)	0.902 (0.869, 0.929)
ResNet 50	0.932 ± 0.048	0.906 ± 0.033	0.920 ± 0.082	1.000	0.929 (0.899, 0.960)	0.872 (0.840, 0.899)	0.804 (0.728, 0.867)	0.895 (0.861, 0.923)
ViT	0.885 ± 0.075	0.884 ± 0.051	0.766 ± 0.151	1.000	0.924 (0.892, 0.955)	0.867 (0.836, 0.895)	0.693 (0.609, 0.769)	0.927 (0.897, 0.951)

The ResNet 50 model was employed for the regression task. The MAE for predicting AL on the test dataset was 0.83 mm (95%CI, 0.72–0.95 mm). The predicted AL and actual AL had a linear relationship with an R^2^ of 0.763 in the ResNet 50 model (Figure 2B). Bland–Altman plots revealed a bias of 0.09 mm, with 95% limits of agreement ranging from −2.2 to 2.3 mm (Figure 2C). Prediction bias of 64.8% of the test dataset was less than 1 mm error (Figure 2D); while a calculation of relative bias revealed that 73.1% of the testing difference was within the range of 5% error and 96.5% within 10% error.

### 3.2 Grad-CAM and model visualization

**Figure 3:**
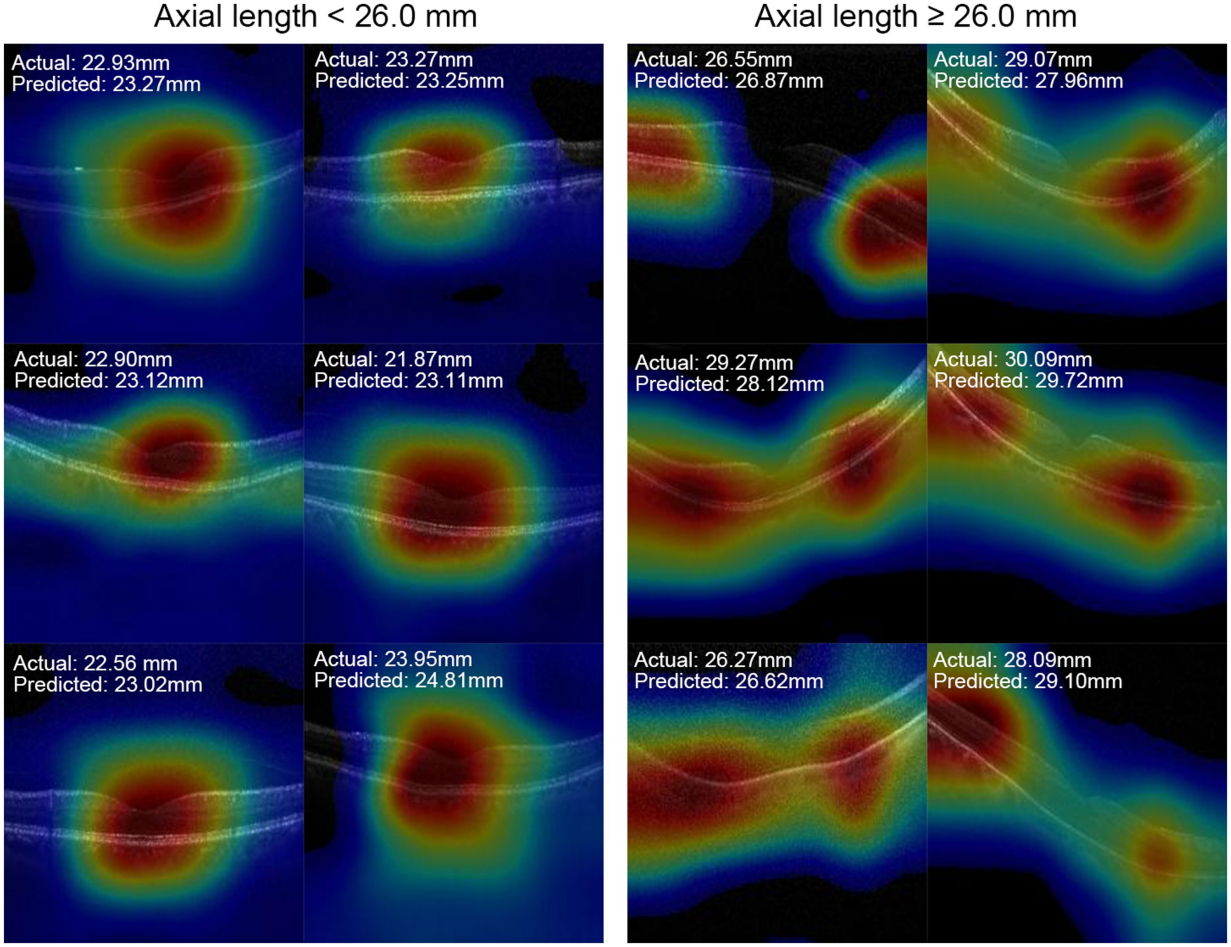
Representative OCT images and their heat map images.

Grad-CAM was used to identify the regions within the original OCT images that the models relied on for their predictions. Figure 3 shows representative OCT images with their corresponding Grad-CAM from the test set, which were correctly predicted. The heat maps revealed that AL-related macular features exhibit a localized pattern in the macula, rather than continuous alterations throughout the entire region. Both the region of retina and choroid were highlighted in the heat maps. For eyes with ALs < 26.0 mm, the CNN models predominantly relied on the curvature and shape of the fovea, whereas for eyes with ALs ≥ 26.0 mm, the models relied on the regions flanking the fovea, where the most obvious retinal curvature changes.

### 3.3 Predicting AL based on retinal thicknesses

The macular thickness of the eyes from the test set was recorded. ROC analyses and linear regression analyses were performed to predict AL based on retinal thickness in different macular regions. The largest AUC value, 0.747, was obtained for CRT. The highest accuracy in distinguishing long AL eyes was 77.8%, achieved by using retinal thickness measurements in the perifoveal (3–6 mm) nasal quadrant (Supplementary material 2). However, both the AUC and accuracy were lower compared to deep learning models that utilized OCT images (*p* < 0.001). Linear regression analyses showed that the MAE values were 1.78 ± 1.25 mm and 1.57 ± 1.29 mm when using CRT and retinal thickness measurements from all nine regions to predict AL, respectively. These biases were also higher than those observed in deep learning models (*p* < 0.001).

## 4 Discussion

The present study demonstrated that deep learning models using macular OCT images can accurately estimate AL and differentiate eyes with long AL. The Grad-CAM analysis revealed that the deep learning models primarily relied on the foveal and adjacent regions, as well as the subfoveal choroid for AL estimation. This deep learning model was designed to estimate the AL based on macular OCT images. This study established a significant association between AL and macular structure, demonstrating the AL-related changes in the macular structure as imaged by OCT. These findings provide a solid foundation for research on the pathogenesis of AL-related structural maculopathy.

Previous studies have used fundus photos to estimate AL via developing deep learning models. Dong et al. (12) and Jeong et al. (17) reported the use of CNN models to estimate AL based on 45 degrees fundus photographs, achieving MAE values of 0.56 mm (95% CI, 0.53–0.61 mm) and 0.90 mm (95% CI, 0.85–0.91 mm), and R^2^ values of 0.59 (95% CI, 0.50–0.65) and 0.67 (95% CI, 0.58–0.87), respectively. Oh et al. (14) developed an AL estimation model using ultra-widefield funds photos with an MAE of 0.74 mm (95% CI, 0.71–0.78 mm) and an R^2^ value of 0.82 (95% CI, 0.79–0.84). However, this study represents the first attempt to estimate AL using macular B-scan OCT images via deep learning. B-scan images provide cross-sectional views of the retina, offering improved visualization of retinal layers and their integrity (18). The theoretical foundation of this study lies in utilizing the potential alterations in macular structure associated with AL elongation to predict AL. Additionally, we also excluded the eyes with any maculopathy to investigate the changes in macular structure before the development of myopic maculopathy in eyes with long AL. In the current study, the MAE was found to be 0.83 mm (95% CI, 0.72–0.95 mm) and the R2 was 0.763 in the regression task, while the classification model achieved an accuracy of 0.872 (95% CI, 0.840–0.899) in identifying eyes with AL ≥ 26.0 mm. These findings suggest that macular structure changes in eyes with long AL occur independently of OCT-detectable myopic maculopathy, which aligns with clinical observations of a higher risk of the prevalence and progression of myopic maculopathy in eyes with longer AL (19, 20).

The results showed that the accuracy of AL estimation using macular OCT images (87.2%) was superior to using retinal thickness measurements alone (77.8%) in the same study sample. This can be attributed to the detailed structural information available in B-scan images (18). The Grad-CAM analysis revealed that for eyes with ALs shorter than 26.0 mm, the deep learning models primarily relied on the fovea, while for eyes with AL greater than or equal to 26.0 mm, the models showed a preference for regions mainly on either side of the fovea. These findings are consistent with a deep learning model for AL estimation using color fundus photos reported by Dong et al. (12). In their study, the heat map analysis demonstrated that eyes with ALs shorter than 26.0 mm predominantly utilized signals from the foveal region in the fundus photos, while those with AL greater than 26 mm primarily relied on signals from the extrafoveal region (12).

Clinical studies have demonstrated that eyes with high myopia, characterized by an AL exceeding 26.0 or 26.5 mm, were more likely to develop traction maculopathy, such as macular hole and maculoschisis (4, 21, 22). Furthermore, Park et al. (23) found that the development of myopic traction maculopathy was associated with the foveal curvature, which were calculated based on the retinal pigment epithelium hyper-reflective line in OCT images including the fovea. Based on the visualization results obtained from our OCT-based AL estimation model, we speculate that the highlighted regions in the heat maps indicate areas where the changes in retinal curvature are most pronounced (24). In addition, our results also suggested that structural changes in the macula caused by axial elongation exhibit a localized pattern, primarily concentrated at the fovea and the areas where the retinal curvature changes the most significantly, rather than displaying continuous alterations throughout the entire region. Besides retina, the choroid from the corresponding regions were also highlighted in the heat maps. Previous studies have reported that AL was negatively associated with choroidal thickness in both young and elderly people (25, 26), indicating the choroidal atrophy with the elongation of AL. These findings can explain the involvement of choroid in the heat maps when predicting AL in this study. These findings will be helpful for further research on the pathogenesis and prevention of AL-related structural maculopathy.

Several limitations should be noted in this study. First, the sample size is relative small. To minimize the impact of potential sources of bias, we specifically enrolled subjects from a solitary ophthalmological clinic and utilized images acquired using the identical imaging machine. Consequently, the recruitment of additional samples was constrained. Advancements in model predictive performance can be expected when more samples are gathered and analyzed. Second, due to the limited number of eyes with short AL in this study, only two groups (whether AL longer than 26.0 mm) were defined in the classification model development. Nevertheless, this limitation is unlikely to undermine the overall findings, as the focus of this study was on the deep learning model’s performance in distinguishing eyes with elongated AL. Third, it is important to note that we excluded eyes with OCT-detectable maculopathy as our aim was to identify AL-specific macular characteristics prior to the onset of myopic maculopathy. Therefore, caution should be exercised when generalizing these findings to eyes with existing maculopathy. Lastly, the current model was developed based on the macular B-scans centered on the fovea by the stellate 6-scan pattern, which scans from 6 different directions. Since OCT B-scans centered on the fovea exhibit the similar imaging pattern, it is very likely that the deep learning model developed in this study would be applicable to macular OCT B-scans scanned by other pattern centered on the fovea or OCT scans from different manufacturers. However, further research and verification are needed to validate the generalization of the model. Additionally, it’s worth noting that this model was developed using adult eyes with a mean age of 69 years. Considering that the macula develops and axial length increases in children and teenagers, additional studies are required to develop models based on younger age groups.

## 5 Conclusion

This study developed a deep learning model using macular OCT images to estimate AL and identify eyes with long AL, achieving good performance. The AL-related macular features exhibit a localized pattern, primarily concentrated in the central fovea and adjacent regions, suggesting that these specific areas may serve as the initial sites for macular alterations caused by AL elongation. These findings have significant implications for further research on the pathogenesis of AL-related structural maculopathy.

## Data availability statement

The raw data supporting the conclusions of this article will be made available by the authors, without undue reservation.

## Ethics statement

The studies involving humans were approved by institutional review board at Beijing Hospital. The studies were conducted in accordance with the local legislation and institutional requirements. The ethics committee/institutional review board waived the requirement of written informed consent for participation from the participants or the participants’ legal guardians/next of kin because the retrospective nature of the study.

## Author contributions

JL: Conceptualization, Data curation, Methodology, Writing – review & editing, Investigation, Validation, Writing – original draft. HL: Conceptualization, Data curation, Investigation, Methodology, Writing – original draft. YoZ: Conceptualization, Investigation, Methodology, Writing – original draft, Software, Validation. YuZ: Conceptualization, Investigation, Methodology, Writing – original draft, Data curation. SS: Conceptualization, Data curation, Investigation, Methodology, Funding acquisition, Supervision, Writing – review & editing. XG: Conceptualization, Data curation, Methodology, Supervision, Writing – review & editing. JX: Conceptualization, Methodology, Supervision, Writing – review & editing, Investigation, Software. XY: Conceptualization, Methodology, Supervision, Writing – review & editing, Data curation, Funding acquisition.
