# Supplementary material for: Deep learning-based estimation of axial length using macular optical coherence tomography images
Source: Front Med (Lausanne). 2023 Nov 17;10:1308923. doi: 10.3389/fmed.2023.1308923 (PMC10693454; doi:10.3389/fmed.2023.1308923)
Supplement: Supplementary file 1 [file Data_Sheet_1.docx]

#### **Supplementary material 1**

#### Deep Residual Network

The ResNet, also known as the Deep Residual Network, is a convolutional neural network introduced by He et al. in 2015. Although AlexNet or VGG have demonstrated impressive performance in computer vision tasks, the network degradation problems become distinguishable when training with a deeper network with more data. By stacking residual blocks containing the skip-connection mechanism, ResNet alleviating the limitation of the number of layers and the gradient vanishing problems in the past models. One key advantage of ResNet over other architectures is its ability to maintain high performance even with an increased number of layers. Furthermore, with the modularized and more light-weighted design of the bottleneck block, ResNet assures flexibility and efficiency. The residual block in ResNet can be expressed as follows:

$$y=H\left( x \right)=F\left( x \right)+x \text{(1)}$$

where the H function and the F function denote the entire residual block and the convolutional block, respectively; x denotes the input of the block; y is the output of the residual block.

The bottleneck design of ResNet is modularized and each residual block consists of two 3x3 convolutional operations or three convolutional operation with 1x1 and 3x3 convolutional kernels. Therefore, the ResNet architecture can be formulated by stacking the bottleneck with different number of layers, which ranges from 18 layers to 152 layers. In this study, we selected ResNet18 and ResNet50 architectures to address the task at hand. This decision was made to avoid overfitting issues that can arise when training large models with limited amounts of data. Although ResNet is certainly robust to the input size, we still restrict the input image as 224x224 for fair comparation with Transformer-based architecture.

#### Vision Transformer

Transformer is a neural network model based on self-attention mechanism. After Vaswani et al. proposed Transformer as a sequence model for machine translation task on 2017, Transformer-based network has become dominant in natural language processing area. The introduction of the Vision Transformer has successfully extended the Transformer architecture to the field of computer vision. It accomplishes this by dividing an image into patches and transforming these patches into linear embeddings, which are then fed as input to a Transformer network. Compared with convolutional networks in vision task, Transformer-based network lacks the inductive bias, translation-invariance and locality, but it can build long-range dependency regardless the distance and only brings quadratic time complexity when the amount of tokens increases. Leveraging pretraining in large-sized dataset like ImageNet, the architecture can learn spatial information of image and surpass convolutional networks. The core design of Transformer is self-attention mechanism, which can be described as follows:

$$Attention\left( Q,K,V \right)=softmax\left( \frac{QK^{T}}{\sqrt{d_{k}}} \right)*V,where Q=K=V \text{(2)}$$

where the input is duplicated as $Q,K,V$ as the input of Attention block and Q,K will firstly calculate the similarity by dot-product to get the attention coefficient and then pass to calculate with V to get self-to-self important tokens; $\sqrt{d_{k}}$ is the token dim to scale the attention coefficient;

To extract the relation between axial length of eyes and macular OCT B-scan, we focus on the macular area and the area from center to both sides. Therefore, we implement Transformer-based architecture which has advantage on long-range dependency modeling. However, Vision Transformer parameterized by the Linear layers is large and tends to get over-fitting when trained with small-sized data. In this study, we adopt the ViT-Base-16-224 as the baseline and reduce the layers of Transformer to 6. This modification helps mitigate the risk of overfitting and ensures better performance on our dataset.
