# Supplementary figures and images for: Deep learning-based estimation of axial length using macular optical coherence tomography images
Source: Front Med (Lausanne). 2023 Nov 17;10:1308923. doi: 10.3389/fmed.2023.1308923 (PMC10693454; doi:10.3389/fmed.2023.1308923)

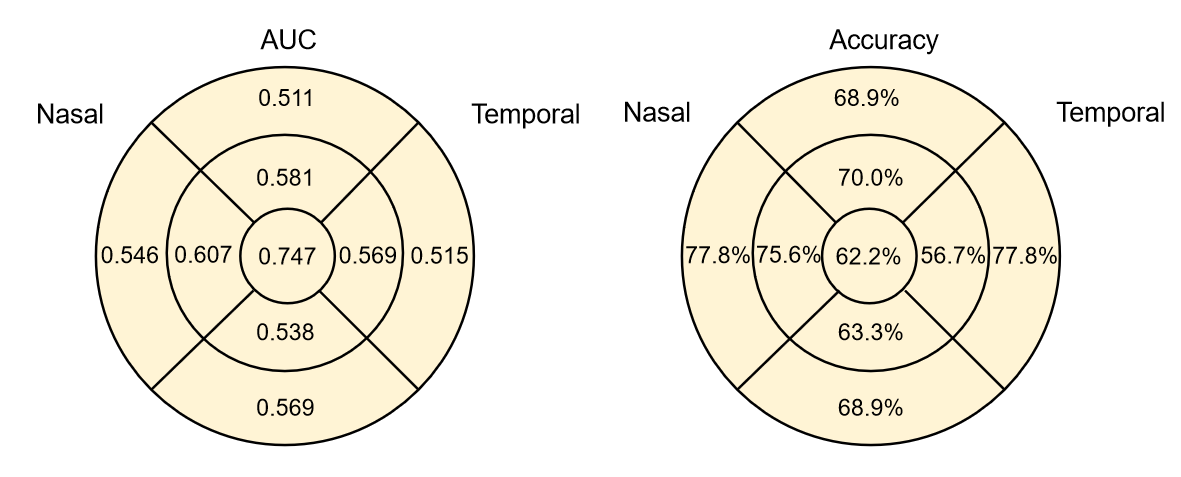

Supplement: Supplementary file 2 [file Image_1.TIF]
